# Predictive genomic markers of response to VEGF targeted therapy in metastatic renal cell carcinoma

**DOI:** 10.1371/journal.pone.0210415

**Published:** 2019-01-25

**Authors:** David D. Stenehjem, Andrew W. Hahn, David M. Gill, Daniel Albertson, Banumathy Gowrishankar, Joseph Merriman, Archana M. Agarwal, Venkata Thodima, Erik B. Harrington, Trang H. Au, Benjamin L. Maughan, Jane Houldsworth, Sumanta K. Pal, Neeraj Agarwal

**Affiliations:** 1 College of Pharmacy, University of Minnesota, Duluth, MN, United States of America; 2 Department of Internal Medicine, Division of Medical Oncology, University of Utah Huntsman Cancer Institute, Salt Lake City, UT, United States of America; 3 Department of Pathology, University of Utah and ARUP Laboratories, Salt Lake City, UT, United States of America; 4 Cancer Genetics Inc., Rutherford, NJ, United States of America; 5 Pharmacotherapy Outcomes Research Center (PORC), College of Pharmacy, University of Utah, Salt Lake City, UT, United States of America; 6 Department of Pathology, Mount Sinai School of Medicine, New York City, NY, United States of America; 7 Department of Medical Oncology & Experimental Therapeutics, City of Hope Comprehensive Cancer Center, Duarte, CA, United States of America; University of Washington, UNITED STATES

## Abstract

**Background:**

First-line treatment for metastatic renal cell carcinoma (mRCC) is rapidly changing. It currently includes VEGF targeted therapies (TT), multi-target tyrosine kinase inhibitors (TKIs), mTOR inhibitors, and immunotherapy. To optimize outcomes for individual patients, genomic markers of response to therapy are needed. Here, we aim to identify tumor-based genomic markers of response to VEGF TT to optimize treatment selection.

**Methods:**

From an institutional database, primary tumor tissue was obtained from 79 patients with clear cell mRCC, and targeted sequencing was performed. Clinical outcomes were obtained retrospectively. Progression-free survival (PFS) on first-line VEGF TT was correlated to genomic alterations (GAs) using Kaplan-Meier methodology and Cox proportional hazard models. A composite model of significant GAs predicting PFS in the first-line setting was developed.

**Results:**

Absence of *VHL* mutation was associated with inferior PFS on first-line VEGF TT. A trend for inferior PFS was observed with GAs in *TP53* and *FLT1* C/C variant. A composite model of these 3 GAs was associated with inferior PFS in a dose-dependent manner.

**Conclusion:**

In mRCC, a composite model of *TP53* mutation, wild type *VHL*, and *FLT1* C/C variant strongly predicted PFS on first-line VEGF TT in a dose-dependent manner. These findings require external validation.

## Introduction

Renal cell carcinoma (RCC) is the sixth highest cause of cancer-related mortality [1]. 25–33% of patients will present with metastatic renal cell carcinoma (mRCC), and an additional 40% of patients who present with localized disease will develop metastases [2, 3]. First-line treatment for mRCC is rapidly evolving as therapies targeting vascular endothelial growth factor (VEGF), MET, mechanistic target of rapamycin (mTOR), and immune checkpoints are currently used. First-line treatments currently approved by the Food and Drug Administration (FDA) include sunitinib, pazopanib, bevacizumab with interferon alpha, sorafenib, temsirolimus, cabozantinib, and nivolumab plus ipilimumab [4]. More changes to first-line treatment are expected to arrive in the near future. Novel combinations of checkpoint inhibitors and VEGF TT (axitinib plus avelumab or pembrozilumab, and bevacizumab plus atezolizumab) are in advanced phases of development and at least some are expected to garner approval in the first-line setting [5]. Despite the availability of so many agents, limited data exists comparing these first-line agents. Thus, selection of a first-line agent is primarily based on comparisons of clinical trial data or anecdotal experiences of individual physicians.

The prognostic risk models, such as International Metastatic Renal Cell Carcinoma Consortium (IMDC), are also useful prognostic tools for mRCC that utilize readily available clinical factors, such as hemoglobin, platelet count, and Karnofsky performance scale, to indirectly reflect the underlying biology of mRCC. These risk models have been validated to predict overall survival prior to different lines of therapy and different classes of drugs [6, 7]. Furthermore, some treatments are only approved for specific IMDC prognostic groups, such as nivolumab plus ipilimumab or temsirolimus. However, they aren’t validated to predict which first-line agent a patient would best respond to among the many available. Genetic biomarkers predictive of differential benefit to first-line treatments are an ideal way to further improve outcomes for mRCC. However, no such biomarkers are routinely used in clinical practice. The purpose of this study was to identify predictive genomic markers of response to VEGF targeted therapy in the first-line setting for mRCC.

## Results

### Patient characteristics and frequency of GAs

**Fig 1:**
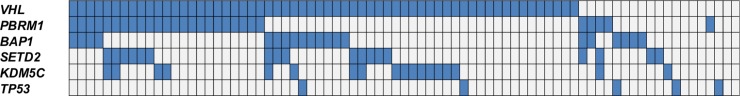
Somatic variants in 79 clear cell mRCC tumors.

A total of 79 patients with mRCC who were treated with first-line VEGF TT and had primary tumor tissue available were included. Patient baseline characteristics are shown in Table 1. For IMDC risk stratification, 60% of patients were intermediate risk and 31% had poor risk disease. The most commonly used first-line treatments were sunitinib (77%) and pazopanib (11%). 30% of patients were previously treated with high-dose interleukin-2, and no patients were previously treated with an immune checkpoint inhibitor. The most common sites of metastatic disease were lung, lymph nodes, bone, and liver. In all patients, GAs in *VHL* (75%) were most common, followed by *PBRM1* (35%), *SETD2* (23%), and *BAP1* (25%), (Table 2, Fig 1**)**. In IMDC intermediate risk patients, *VHL* (72%), *PBRM1* (40%), *SETD2* (28%), and *KDM5C* (26%) were the most prevalent GAs.

**Table 1:** Baseline patient characteristics.

All Patients	N = 79
**Age, y (%)**	
Median (IQR)	61 (55–70)
**Gender, n (%)**	
Male	56 (71)
**Race, n (%)**	
White	70 (89)
Hispanic	3 (4)
Other	6 (8)
**IMDC risk criteria, n (%)**	
Favorable	7 (9)
Intermediate	47 (60)
Poor	24 (31)
**Prior cytokine-based immunotherapy, n (%)**	
Yes	24 (30)
**First line treatment, n (%)**	
Sunitinib	61 (77)
Sorafenib	6 (8)
Pazopanib	9 (11)
Bevacizumab	3 (4)
**Sites of Metastases, n (%)**	
Lung	56 (71)
Lymph nodes	36 (46)
Bone	29 (37)
Liver	17 (22)
Peritoneum	2 (3)
Brain	8 (10)
Other	39 (49)

**Table 2:** Frequency of gene mutations and germline FLT1 allelic variants in all patients and IMDC intermediate risk patients.

Mutations identified	All Patients n = 79	IMDC Intermediate n = 47	IMDC poor n = 24	IMDC favorable n = 7
***VHL***	60 (76%)	34 (72%)	19 (79%)	6 (86%)
***PBRM1***	28 (35%)	19 (40%)	7 (29%)	2 (29%)
***SETD2***	18 (23%)	13 (28%)	5 (21%)	0 (0%)
***BAP1***	20 (25%)	10 (21%)	7 (29%)	3 (43%)
***KDM5C***	18 (23%)	12 (26%)	5 (21%)	1 (14%)
***MAGEC1***	13 (16%)	6 (13%)	5 (21%)	2 (29%)
***MTOR***	12 (15%)	7 (15%)	5 (21%)	0 (0%)
***ROS1***	7 (9%)	5 (11%)	1 (4%)	1 (14%)
***TP53***	5 (6%)	3 (6%)	2 (8%)	0 (0%)
***FLT1* (rs9582036)** *				
A/A	46 (58%)	26 (55%)	15 (63%)	4 (57%)
A/C	26 (33%)	15 (32%)	9 (38%)	2 (29%)
C/C	7 (9%)	6 (13%)	0 (0%)	1 (14%)
**Composite of *VHL* wildtype, mutated *TP*53, and *FLT1* C/C**				
Zero	54 (68%)	31 (66%)	17 (71%)	5 (71%)
One	20 (25%)	11 (23%)	7 (29%)	2 (29%)
Two or three	6 (8%)	5 (11%)	0 (0%)	0 (0%)

*(A/A or A/C vs C/C); A/A, A/C, C/C represent the genotype of FLT1/VEGFR1 SNP (rs9582036).

### Correlation of GAs and progression-free survival on first-line VEGF TT in all patients

**Fig 2:**
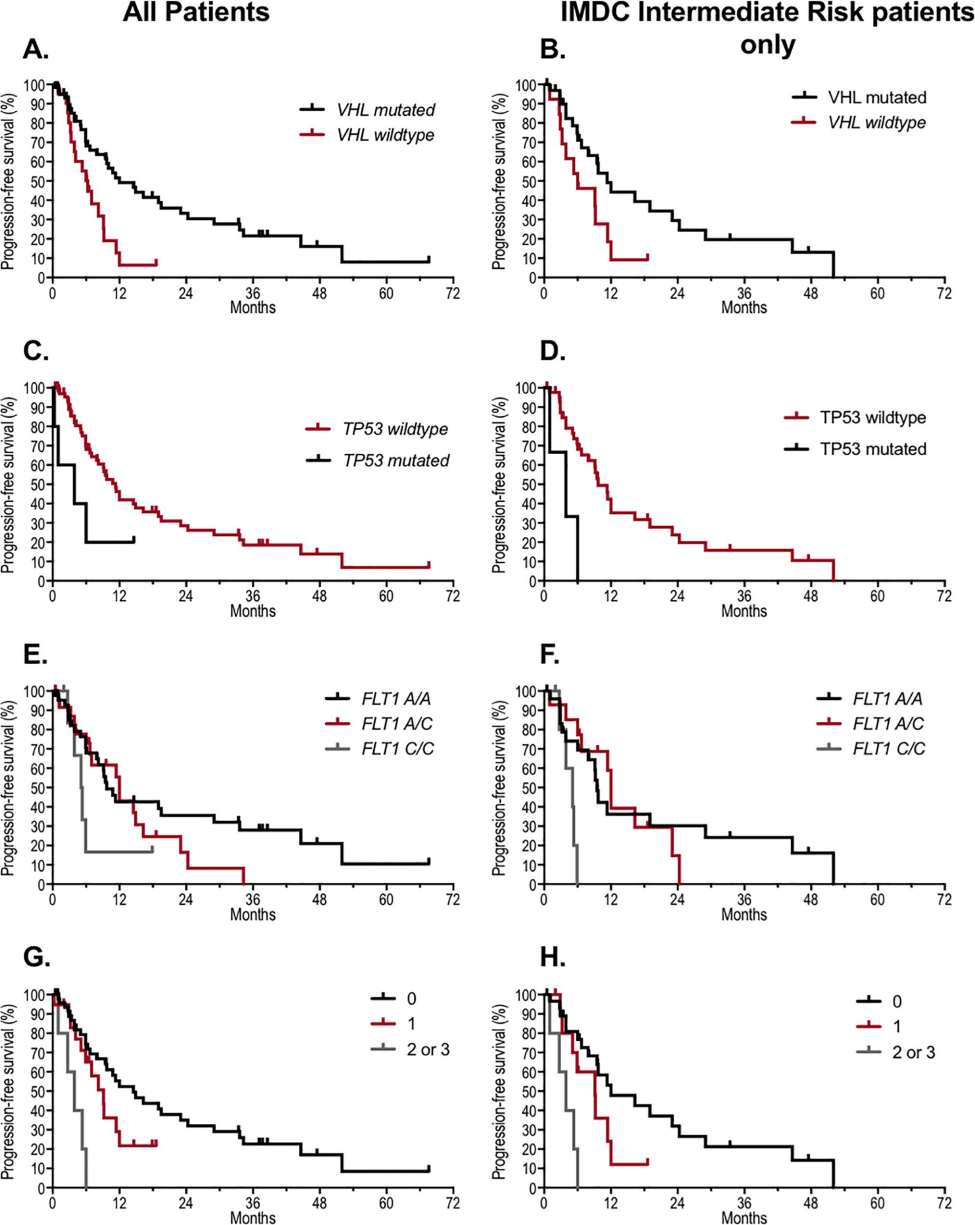
Progression-free survival on first line therapy. *VHL* (A, B), *TP53* (C, D), *FLT1* (E, F) variants, and composite of *VHL* wildtype, *TP53* mutated, and *FLT1* C/C (G, H) in all patients (left panel) and IMDC intermediate risk patients only (right panel).

*VHL* mutations were associated with improved PFS (HR 0.41, 95% CI 0.21–0.82; p = 0.007) (Table 3, Fig 2A**)**. *TP53* mutations demonstrated a trend towards shorter PFS in the first-line setting (3.9 vs. 11.3 months, HR 2.61, 95% CI 0.78–6.57; p = 0.059), (Table 3, Fig 2C**)**. *PBRM1*, *SETD2*, *BAP1*, *KDM5C*, *MAGEC1*, and *mTOR* mutations were not associated with significant differences in PFS compared to wild type. A trend for inferior PFS was observed in patients with the *FLT1* C/C variant (5.2 months) compared to the A/A variant (9.7 months, p = 0.074) and the A/C variant (12 months, p = 0.17) respectively (Table 3, Fig 2E). After correction for IMDC prognostic criteria in the Cox proportional hazard models, *VHL* mutations remained a significant predictor of improved PFS in the first-line setting (HR 0.45, 95% CI 0.23–0.89; p = 0.022).

**Table 3:** Median progression-free survival by gene variants in all patients and in IMDC intermediate risk patients.

Gene	All Patients				IMDC Intermediate risk criteria patients			
	n = 79	PFS (mos)	HR (95% CI)	Log-Rank	n = 47	PFS (mos)	HR (95% CI)	Log-Rank
***VHL***								
Mutation	60	14.5	0.41 (0.21–0.82)	***0*.*0070***	34	11.3	0.43 (0.20–0.97)	***0*.*029***
Wildtype	19	7.0			13	6.0		
***PBRM1***								
Mutation	28	14.9	0.77 (0.42–1.40)	0.40	19	12.0	0.59 (0.27–1.21)	0.15
Wildtype	51	9.2			28	9.1		
***SETD2***								
Mutation	18	13.5	1.01 (0.47–1.96)	0.98	13	13.5	0.79 (0.33–1.71)	0.57
Wildtype	61	9.7			34	9.5		
***BAP1***								
Mutation	20	8.2	1.13 (0.59–2.08)	0.69	10	9.1	1.13 (0.47–2.45)	0.76
Wildtype	59	11.3			37	9.7		
***KDM5C***								
Mutation	18	11.4	0.98 (0.47–1.87)	0.95	12	11.4	0.91 (0.36–2.01)	0.82
Wildtype	61	9.7			35	9.2		
***MAGEC1***								
Mutation	13	7	0.80 (0.30–1.76)	0.60	6	6.7	0.83 (0.24–2.14)	0.72
Wildtype	66	10.8			41	9.7		
***mTOR***								
Mutation	12	7.3	1.80 (0.84–3.51)	0.10	7	9.2	1.12 (0.37–2.73)	0.82
Wildtype	67	11.4			40	9.7		
***ROS1***								
Mutation	7	7.7	1.17 (0.40–2.70)	0.71	5	7.3	1.73 (0.51–4.50)	0.82
Wildtype	72	10.8			42	9.7		
***TP53***								
Mutation	5	3.9	2.61 (0.78–6.57)	0.059	3	3.9	4.73 (1.07–15.01)	***0*.*007***
Wildtype	74	11.3			44	9.7		
***FLT1*** (rs9582036)								
A/A	46	9.7	A/A vs A/C: 0.71 (0.38–1.36)	0.29	26	9.5	A/A vs A/C: 0.92 (0.46–2.46)	0.81
A/C	26	12	A/C vs C/C: 0.54 (0.21–1.66)	0.17	15	12	A/C vs C/C: 0.20 (0.06–0.71)	***0*.*0012***
A/A or A/C	73	11.3	A/A or A/C vs C/C: 0.44 (0.19–1.30)	0.08	41	11.3	A/A or A/C vs C/C: 0.19 (0.06–0.63)	***0*.*0010***
C/C	7	5.2	C/C vs A/A: 2.61 (0.86–6.56)	0.074	6	5.1	C/C vs A/A: 5.52 (1.40–17.4)	***0*.*0058***
**Composite of *VHL* wildtype, mutated *TP53*, and *FLT1* C/C**								
0	54	14.5	1 vs 0: 1.78 (0.85–3.57)	0.11	31	12	1 vs 0: 2.09 (0.82–4.99)	0.12
1	20	9.1	2 or 3 vs 1: 3.83 (1.18–10.88)	***0*.*0052***	11	9.1	2 or 3 vs 1: 3.80 (1.08–12.55)	***0*.*038***
2 or 3	5	3.9	2 or 3 vs 0: 6.83 (2.17–18.26)	***0*.*0001***	5	3.9	2 or 3 vs 0: 7.93 (2.31–24.64)	***0*.*0018***

MOS, months; PFS, progression-free survival, HR, hazard ratio

### Developing a composite model of predictive GAs for response to first line VEGF TT for all patients

Since *VHL* wild type, mutated *TP*53, and *FLT1* C/C SNP were associated with a trend towards shorter PFS (Table 3), we hypothesized that a composite model utilizing these 3 GAs would serve as a stronger predictive biomarker for response to first-line VEGF TT in clear cell mRCC. The composite model was associated with inferior PFS in a dose-dependent manner (Table 3, Fig 2G). Patients with 2 or 3 GAs had PFS of 3.9 months, whereas those harboring 1 GA had PFS of 9.1 months (HR 3.83, 95% CI 1.18–10.88, p = 0.005). In comparison to the PFS of 3.9 months seen in those with 2 or 3 GAs, patients with no GAs had superior PFS at 14.5 months (HR 6.83, 95% CI 2.17–18.26, p = 0.01). When controlling for IMDC risk category in a Cox proportional hazard model, the composite model was still predictive of inferior PFS in a dose-dependent manner (Table 4). Finally, presence of 1 or more GAs in the composite model was prognostic for inferior overall survival (OS) (Table 4).

**Table 4:** Cox proportional hazard model for PFS and overall survival by IMDC risk criteria and sum of *VHL* wildtype, *TP53* mutated, and *FLT1* C/C genotype (rs9582036).

	Progression-free Survival		Overall Survival	
	Hazard ratio, 95% CI		Log-Rank		Hazard ratio, 95% CI		Log-Rank	
***IMDC Risk Criteria***				
Favorable	ref		ref	
Intermediate	4.76 (1.41–29.68)	***0*.*0084***	2.84 (0.83–17.80)	0.10
Poor	6.26 (1.70–40.41)	***0*.*0039***	6.48 (1.76–41.79)	***0*.*0031***
**Composite of *VHL* wildtype, mutated *TP53*, and *FLT1* C/C**				
0	1 vs 0: 1.70 (0.81–3.42)	0.15	1 vs 0: 2.36 (1.11–4.80)	***0*.*026***
1	2 or 3 vs 1: 3.76 (1.13–11.03)	***0*.*032***	2 or 3 vs 1: 2.16 (0.48–7.21)	0.28
2 or 3	2 or 3 vs 0: 6.40 (2.00–17.57)	***0*.*0033***	2 or 3 vs 0: 5.11 (1.15–16.41)	***0*.*035***

## Discussion

Numerous targeted therapies are available for first-line treatment of mRCC, and more are expected to receive approval in the near future. Yet, limited data on genetic biomarkers exist, and no biomarkers are currently used in the clinic to guide treatment selection in mRCC. In our study, patients with wild type *VHL* had shorter PFS in response to VEGF targeted therapies compared to those with GAs in *VHL*. Furthermore, GAs in *TP53* and the *FLT1* C/C SNP were associated with a trend towards shorter PFS. A composite model using wild type *VHL*, mutated *TP53*, and *FLT1* C/C was predictive of response to first-line VEGF targeted therapies in a dose-dependent manner. Since the composite model was predictive of inferior PFS when controlling for IMDC risk group, it could be used to complement a clinical prognostication tool, such as the IMDC risk score.

Comprehensive characterization of stage I-IV RCC by The Cancer Genome Atlas (TCGA) demonstrated that the 8 most frequent mutations in RCC are: *VHL*, *PBRM1*, *SETD2*, *KDM5C*, *PTEN*, *BAP1*, *MTOR*, and *TP53* [8]. Biallelic inactivation of *VHL* is common in RCC. *VHL* encodes a protein that ubiquitinates HIF to mark it for proteasome degradation. Increased levels of HIF result in increased expression of its downstream targets, including VEGF [9]. To date, studies of whether mutational status of *VHL* is predictive of response to VEGF targeted therapy produced mixed results [10–13]. In a retrospective analysis of 123 patients treated with VEGF targeted therapy, loss of function mutations in *VHL* were associated with improved response rates compared to wild-type *VHL* (52% vs. 31%, p = 0.04) [10]. However, *VHL* mutation/methylation status did not correlate with response rates or PFS in an analysis of 78 patients from a clinical trial evaluating pazopanib in mRCC [11]. *TP53* encodes a well-known tumor suppressor protein and is a known prognostic biomarker for breast cancer, squamous cell carcinoma of the head and neck, and prostate cancer [14–16]. In clear cell RCC, genomic alterations in *TP53* are a poor prognostic marker for overall survival (OS) [17]. A recent study also found increasing frequency of *TP53* mutations after first-line VEGF TT, which suggests that *TP53* may play a role in resistance [18]. *FLT1* encodes the VEGFR and is the only validated, predictive, germline biomarker for response to VEGF TT in mRCC. An initial screen of 138 SNPs in patients treated with bevacizumab for either metastatic pancreatic or RCC found that only rs9582036 was predictive of PFS in mRCC [19]. They then studied *FLT1* in patients with mRCC who were treated with first-line sunitinib and found the C/C variant was predictive of inferior RR, PFS, and OS [20, 21]. In our cohort, *FLT1* C/C had a trend towards significance in the entire cohort and did predict inferior PFS in IMDC intermediate risk patients.

Recently, a few studies have reported the frequency of mutations in only mRCC, instead of all stages of RCC [12, 13, 22]. In our cohort, the incidence of *VHL* mutations (75%, 71–83%) and *TP53* mutations (6%, 8–11%) was similar to previously reported studies. More recognition has been given to the potential role of *PBRM1*, *BAP1*, *SETD2*, and *KDM5C* mutations in RCC. In a study of 111 patients treated with first-line sunitinib by Hsieh et al., they found that mutant *KDM5C* was predictive of superior PFS compared to wild type (20.6 months vs. 8.3 months, p = 0.03) [13]. In a separate study of 95 patients treated with first-line VEGF TT, time-to-treatment-failure significantly differed by *PBRM1* and *BAP1* mutation status, no significant difference was seen with *KDM5C* [22]. In our study, we did not see a significant difference in PFS associated with mutations in *PBRM1*, *BAP1*, or *KDM5C*. To date, each study of first-line VEGF TT in mRCC, including ours, had a similar number of patients, was retrospective, and produced differing results. These findings suggest that larger and ideally prospective genetic biomarker studies are needed to validate the findings of these multiple small studies. Prospective clinical trials for novel treatments in mRCC need to include predictive biomarker studies that may help personalize first and second-line treatment for mRCC.

Limitations of our study include its retrospective nature, limited cohort size with few IMDC favorable risk patients, and use of multiple VEGF targeted therapies. Unlike PFS and OS, the data on objective responses were not reliably collected in this retrospective analysis, and hence correlation with objective responses with the underlying GAs was not performed. While use of multiple VEGF TT may introduce heterogeneity into our outcomes, it also is more realistic for eventual use in the real world. In regards to IMDC risk group, few of our patients were IMDC favorable risk. While this was due to random selection, it would have been interesting to assess GAs and response to VEGF TT in more patients with IMDC favorable risk disease because favorable risk disease had improved response to VEGF TT in CheckMate 214. Future studies based on the results of our data could include: validation of the composite model while accounting for IMDC risk group, use of circulating tumor DNA NGS to assess if the composite model remains significant, and use of ctDNA to assess the frequency of the eight significant mutations in RCC.

## Materials and methods

From an institutional database, patients diagnosed with metastatic clear cell predominant RCC, hereafter mRCC, between the years 2000–2013 who were treated with first-line VEGF TT and had primary tumor tissue available from nephrectomy for genomic analysis were included. A retrospective chart review was conducted to determine first-line treatment, duration of response, and IMDC risk criteria, and sites of metastases. For clarity, a predictive biomarker is one that predicts a differential response to specific treatments; whereas, a prognostic biomarker is one that yields information regarding a patient’s overall cancer outcome. Genomic DNA was extracted from macro-dissected FFPE sections of tumors ensuring >70% tumor burden. Gain/loss was evaluated by array-CGH (Agilent 4x180K) and differential (≥25/30%) copy number alterations (CNAs) were assessed using Nexus Copy Number Algorithm (BioDiscovery, Inc.). CNAs with >25% difference for weighted average frequency (WAF) and p<0.05 were considered significant [23]. Nucleotide variants were detected by massively parallel sequencing using a custom hybrid capture panel comprising 76 RCC-relevant mutated genes (covering coding exons and splice junctions) and 7 prognostic SNPs (S1 and S2 Tables), on a MiSeq (Illumina) to an average depth of ~300x. CLC Biomedical Genomic Workbench (Qiagen) was used for variant detection and Annovar was used for variant annotation. A schematic representing sequencing data analysis steps is provided in S1 Fig. Variants with a VAF (variant allele frequency) > 5% were considered further. The study was approved by the Institutional Review Board at the University of Utah (IRB# 00067518) and written consent was obtained from all patients.

### Statistical analysis

The PFS was described using the Kaplan-Meier analysis and compared by genomic variants using the log-rank test. Cox proportional hazard models were created combining risk criteria and mutations status.

## Conclusion

A composite model of tumor *TP53* mutation, wild type *VHL*, and *FLT1* C/C SNP is predictive of outcomes to treatment with VEGF TT in the first-line setting in a dose-dependent manner. Patients harboring tumor genomic markers predicting poor outcomes to VEGF targeted therapy may be candidates for agents targeting primarily non-VEGF pathways, such as checkpoint inhibitors, c-MET inhibitors, a combination of VEGF-TKI plus checkpoint inhibitors, or clinical trials. These results are hypothesis-generating and need external validation.

## Supporting information

S1 Table Selected genes included in panel for analysis. (DOCX)

S2 Table SNPs tested in analysis. (DOCX)

S1 Fig Schematic diagram depicting the bioinformatic flow for somatic variant identification. (TIFF)
