# Supplementary material for: Predictive genomic markers of response to VEGF targeted therapy in metastatic renal cell carcinoma
Source: PLoS One. 2019 Jan 25;14(1):e0210415. doi: 10.1371/journal.pone.0210415 (PMC6347137; doi:10.1371/journal.pone.0210415)
Supplement: S1 Table — (DOCX) [file pone.0210415.s001.docx]

**S1 Table.** **Selected genes included in panel for analysis.**

| **Genes tested (76)** | | | |
| --- | --- | --- | --- |
| ABL1 | EGFR | NDRG1 | SFXN4 |
| AFF1 | EPHB4 | NPNT | SMO |
| AKT1 | ERBB2 | PBRM1 | SMOX |
| AKT2 | FGFR1 | PCK1 | SOX4 |
| ALK | FLT3 | PCNA | SPRED1 |
| ARID1A | FYN | PDGFRB | SRC |
| AXL | GLI3 | PGLYRP3 | STAG2 |
| BAP1 | GOLGA5 | PIK3CA | TET2 |
| BMPR1B | GUSB | PIK3CG | TNC |
| BRAF | HIF1A | PTCH1 | TP53 |
| BRINP2 | HSP90AA1 | PTEN | TSC1 |
| CARD11 | IFNB1 | RAF1 | TSC2 |
| CCDC120 | ING4 | RALGAPA1 | UBE2D1 |
| CCND2 | JAK2 | RAPGEF2 | VHL |
| CDH4 | KDM5C | RASSF2 | ZFPM2 |
| CUL1 | KIT | RBAK | ZNF800 |
| DAXX | MAGEC1 | RHEB |  |
| DFNA2 | MAPK14 | ROS1 |  |
| DIO2 | MET | SCARB2 |  |
| DST | MTOR | SETD2 |  |

Red = frequently mutated genes in RCC.

Purple = actionable drug targets.
