# Supplementary material for: Predictive genomic markers of response to VEGF targeted therapy in metastatic renal cell carcinoma
Source: PLoS One. 2019 Jan 25;14(1):e0210415. doi: 10.1371/journal.pone.0210415 (PMC6347137; doi:10.1371/journal.pone.0210415)
Supplement: S2 Table — (DOCX) [file pone.0210415.s002.docx]

**S2 Table.** **SNPs tested in analysis.**

| **SNPs (7)** |
| --- |
| rs7121 (GNAS1) |
| rs11549465 (HIF1A) |
| rs1126647 (IL8) |
| rs4073 (IL8) |
| rs11762213 (MET) |
| rs699947 (VEGFA) |
| rs9582036 (VEGFR1) |
