# Supplementary figures and images for: Predictive genomic markers of response to VEGF targeted therapy in metastatic renal cell carcinoma
Source: PLoS One. 2019 Jan 25;14(1):e0210415. doi: 10.1371/journal.pone.0210415 (PMC6347137; doi:10.1371/journal.pone.0210415)

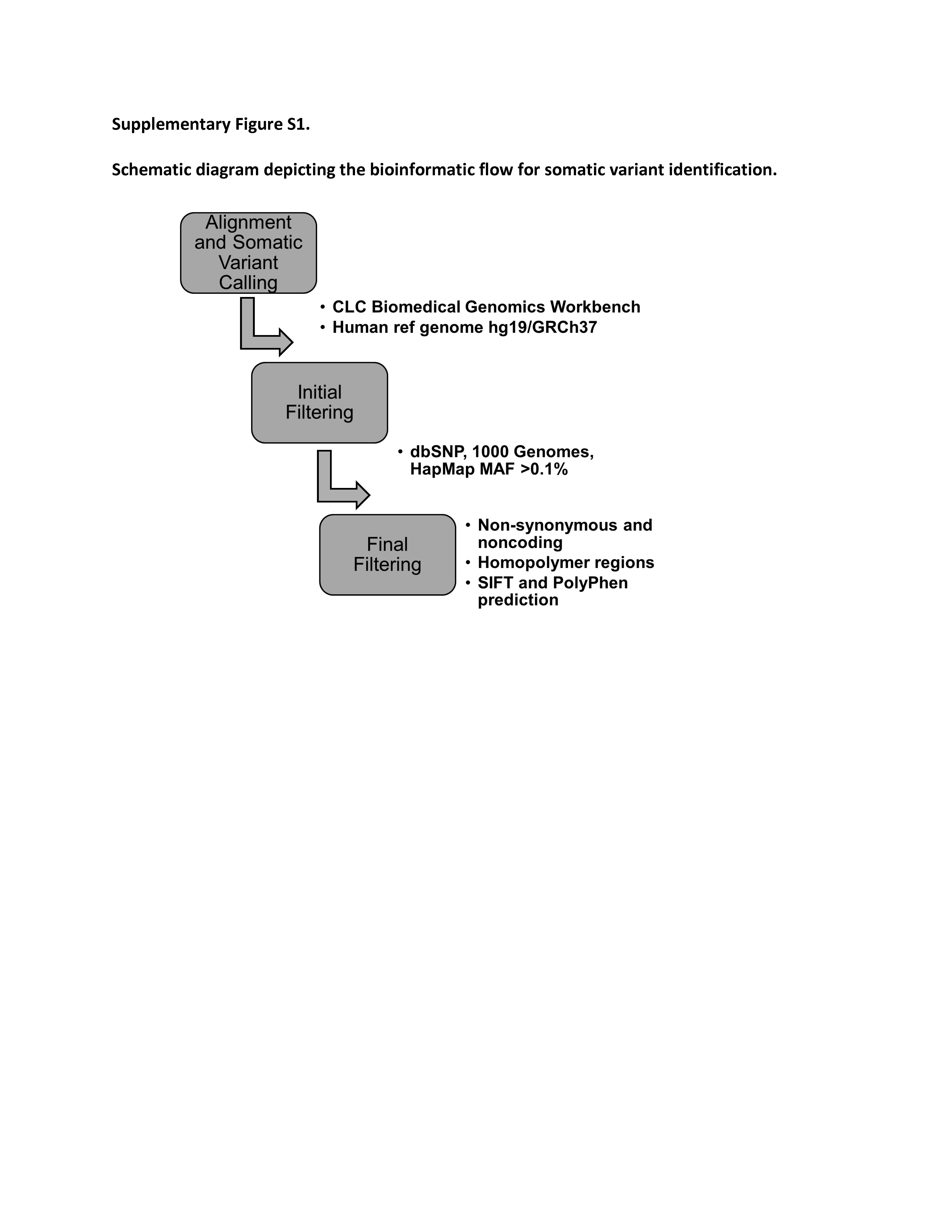

Supplement: S1 Fig — (TIFF) [file pone.0210415.s003.tiff]
